# Recombinant SARS-CoV-2 Delta/Omicron BA.5 emerging in an immunocompromised long-term infected COVID-19 patient

**DOI:** 10.1038/s41598-024-75241-3

**Published:** 2024-10-28

**Authors:** Ignacio Garcia, Jon Bråte, Even Fossum, Andreas Rohringer, Line V. Moen, Olav Hungnes, Olav Fjære, Kyriakos Zaragkoulias, Karoline Bragstad

**Affiliations:** 1 Department of Bacteriology, Norwegian Institute of Public Health, Oslo, Norway; 2 Department of Virology, Norwegian Institute of Public Health, Oslo, Norway; 3 Section for Infectious Diseases, Department of Internal Medicine, Levanger Hospital, Nord-Trøndelag Hospital Trust, Levanger, Norway; 4 Section for Medical Microbiology, Department of Laboratory Medicine, Levanger Hospital, Nord-Trøndelag Hospital Trust, Levanger, Norway; 5 Department of Medical Microbiology, St Olavs Hospital, Trondheim University Hospital, Trondheim, Norway

**Keywords:** SARS-CoV-2, Recombinant, Immunocompromised, In-patient recombination event, Delta, Omicron, Molecular evolution, Genetic variation, Viral genetics

## Abstract

The emergence of the SARS-CoV-2 virus led to a global pandemic, prompting extensive research efforts to understand its molecular biology, transmission dynamics, and pathogenesis. Recombination events have been increasingly recognized as significant contributor to the virus’s diversity and evolution, potentially leading to the emergence of novel strains with altered biological properties. Indeed, recombinant lineages such as the XBB variant and its descendants have subsequently dominated globally. Therefore, continued surveillance and monitoring of viral genome diversity are crucial to identify and understand the emergence and spread of novel strains. Through routine genomic surveillance of SARS-CoV-2 cases in Norway, we discovered a SARS-CoV-2 recombination event in a long-term infected immunocompromised COVID-19 (coronavirus disease) patient. A deeper investigation showed several recombination events between two distinct lineages of the virus, namely AY.98.1 and BA.5, that resulted in a single novel recombinant viral strain with a unique genetic signature. Our data is consistent with the presence of several concomitant recombinants in the patient, suggesting that these events occur frequently in vivo. This study underscores the importance of continued tracking of viral diversity and the potential impact of recombination events on the evolution of the SARS-CoV-2 virus.

## Introduction

The emergence of the severe acute respiratory syndrome coronavirus 2 (SARS-CoV-2) caused a global pandemic that affected millions of people worldwide, with significant impacts on public health^1^, the economy^2^, and social welfare^3^. The high rate of transmission^4^ and the ability of the virus to cause severe respiratory illness^5^ have prompted extensive research efforts to understand its biology and evolution. One crucial aspect of this research is investigating the genetic variability of the virus, including the potential for recombination events, as these can lead to the emergence of new viral strains with altered virulence and transmission characteristics.

Recombination occurs when two or more different viral strains infect the same host cell, allowing the exchange of genetic material between the viruses. In coronaviruses, these events are primarily driven by the RNA-dependent RNA polymerase, which can switch between viral templates during genome replication^6^. This can result in the formation of chimeric viruses that contain genetic material from two or more viral strains^7^. The emergence of recombinant viruses has been increasingly recognized as a significant contributor to SARS-CoV-2 diversity and evolution.

While recombinant SARS-CoV-2 viruses were observed during the first years of the COVID-19 pandemic, these variants did not circulate widely in the population^8,9^. However, as the number of infections rose with the spread of the Omicron variant, there was also an increase in observed recombinant strains, including the emergence of the XBB lineage, a recombination between the two BA.2.75 subvariants, BJ.1.1 and BM.1.1.1^10^, which initially resulted in extensive transmission in Singapore, India, and elsewhere in the fall of 2022^11^. By the spring of 2023, subvariants of XBB had become dominant globally, demonstrating how recombination events can contribute to viral fitness and transmissibility.

Chronic SARS-CoV-2 infections in immunocompromised individuals are known to accelerate viral mutagenesis, and significant mutations within the spike protein have been observed in these patients^12,13^. Moreover, the prolonged persistence of the infection in these patients provides a favorable time window for recombination to occur if the patient is exposed to other variants. Indeed, several recombinants have been identified to occur in immunocompromised patients^8,14^.

In this study, we report the identification of a recombinant SARS-CoV-2 virus in a long-term infected COVID-19 patient. During our surveillance of SARS-CoV-2, we identified the emergence of a recombinant strain between two distinct lineages, AY.98 and BA.5, resulting in a novel viral strain. We gathered and characterized additional samples from the same patient before and after the recombination event. Deep sequencing of all the sequences suggests that recombination events occur frequently in vivo, providing further evidence of the need for continued surveillance and monitoring of viral diversity in immunocompromised patients. Our findings underscore the importance of understanding the molecular mechanisms of recombination and the potential impacts of recombination events on the evolution and emergence of novel strains of SARS-CoV-2.

## Methods

### Sample extraction and sequencing

All the samples were extracted and processed using the Swift Amplicon SARS-CoV-2 Panel (Integrated DNA Technologies). The samples were sequenced on an Illumina NovaSeq platform at the Norwegian Sequencing Centre (NSC) NorSeq.

### Generation of SARS-CoV-2 consensus sequences

SARS-CoV-2 consensus sequences were generated using the “Covid-seq” pipeline developed by the NSC (https://github.com/nsc-norway/covid-seq). Briefly, PCR primers used during library preparation were removed using NSCTrim (https://github.com/nsc-norway/NSCtrim). Then, sequencing adapters, poorly called nucleotides, and overall low-quality reads and adapters were removed using fastp^15^.

Next, the high-quality-trimmed reads were mapped to the *Wuhan-Hu-1* reference genome (NC_045512.2) using Bowtie2^16^. Consensus sequences were generated from the resulting mapping files using samtools, mpileup^17^ and iVar^18^ with a minimum depth threshold of 10 for calling a nucleotide.

### Noise calculation

We define noise as the sum of the ratios of all the nucleotides minus the ratio of the most frequent nucleotide (i.e., the one called in the consensus sequence). To calculate the noise of the samples we developed a tool called NoisExtractor (https://github.com/garcia-nacho/NoisExtractor). NoisExtractor uses indexed bam files as inputs, and for each position of the genome, it outputs the noise, depth, the nucleotide with highest frequency and the nucleotide with the second highest frequency and their frequencies respectively.

### Identification of coinfections/contaminations

As part of the sequencing routines at NIPH, a quality control is performed for each sample. In this analysis low-quality samples and individual samples containing more than one virus are flagged, as this could indicate a contaminated sample or a coinfection at the patient level. To do this analysis, we developed a machine learning model. This model is based on linear regression in which noise-related parameters (e.g., mean and standard deviation of noise across the genome, binned number of positions with noise, etc) and depth and coverage-related parameters (e.g., binned number of missing positions, average depth, etc) were used to classify a sample as low-quality, high-quality or contaminant. To train the classification model, we used a subset of 1846 manually curated samples that were assigned into 4 different classes: "high-quality-high-contamination", "high-quality-low-contamination", "high-quality-no-contamination" and "low-quality”. The code to perform the quality control and the trained model is available at here: https://github.com/folkehelseinstituttet/FHI_SC2_Pipeline_Illumina.

### Extraction of sequences for the major and minor variants

Once a possible contamination or coinfection is identified, the sequence of the major variant (most abundant variant) was generated by concatenating the nucleotides with the highest frequency at each position of the genome. To generate the sequence of the minor variant (second most abundant variant), the nucleotides in which the noise of the sequence was higher than 0.1 were replaced. The nucleotide that replaced the nucleotide with highest frequency (major) was the one with the second highest frequency (minor). We implemented the extraction of sequences by parsing the output of NoisExtractor in R.

### Identification of recombinant sequences

To identify recombinant sequences, we developed PrecFinder (https://github.com/garcia-nacho/Precfinder). For each single mutation in a sequence, PrecFinder calculates the Bayes’ probability of the virus belonging to a particular Pangolin lineage based on the distribution of mutations in different lineages. As the ratios of the different mutations in the virus continue to evolve, the probability is calculated based on a database of sequences that is regularly updated.

To find which sequences are recombinants, PrecFinder uses a 1D-convolutional neural network model. The model consists of three 1D-convolutional neural network layers (1D-CNN) followed by a 1D-MaxPooling layer. The three 1D-CNN have 64, 32 and 12 filters and kernel sizes of 5, 3 and 3 nucleotides respectively. The pool sizes of all the 1D-MaxPooling layers were set to 2. Then, two feed-forward layers with 24 and 12 layers, respectively, were included. Finally, a softmax classification layer outputs the score to classify the sample. The input of the model consists of a Bayes’ probability matrix of *n* by *m* dimensions. Where *n* is the number of unique lineages present on the database and *m* is the maximum number of mutations present in at least one sequence of the database. To train the model, we used binary cross-entropy as loss function, adagrad as optimizer and a batch size of 128. The training of the model was scheduled for 60 epochs but it was early-stopped if there was no improvement on the accuracy after eight epochs. The weights of the model with highest accuracy were saved. Moreover, F1, precision and recall were calculated. As training set, we used the sequences present in the database which consists of a synthetic set of recombinant sequences that were generated using the sequences present in the database. Sequences assigned to different lineages were recombined in silico through one, two or three breaking-points randomly selected in the genome. Moreover, we augmented the dataset through the reordering the *n* rows of the training set. The model was implemented and trained using Keras^19^ and TensorFlow v2.8^20^.

Recombinant sequences were also identified using the program sc2rf (https://github.com/lenaschimmel/sc2rf).

### Lineage assignments

SARS-CoV-2 lineage assignment was performed using Pangolin (PUSHER-v1.18.1.1)^21^ and Nextclade (v2.7.0)^22^ and the mutations at nucleotide and amino acid levels were identified using Nextclade.

To identify the AY.98.1 and BA.5 specific mutations, 2000 AY.98.1 and BA.5 sequences were downloaded from NCBI GenBank using cov-sampler^23^. Sequences with low-quality and/or wrong lineage assignment according to Nextclade were removed, and the mutations present in the remaining sequences were extracted using Nextclade. Based on the frequency of the mutations present on the AY.98.1 and BA.5 lineages, the mutations present in our sequences were classified either as AY.98.1-specific, BA.5-specific, or other, where other means that the mutation is not found on any of the lineages or that it can be found in both. All plots to visualize Pangolin lineages were generated in R^24^ using the library ggplot2^25^.

### Routine quality control

For each of the samples sequenced, we assessed the noise (see “Methods”: “Noise calculation”), the presence of coinfections/contaminations (see “Methods”: “Identification of coinfections/contaminations”), and the presence of recombinants (see “Methods”: “Identification of recombinant sequences”).

### In silico mix of viral lineages

To estimate the noise expected from a mixture of viruses, we created an R-Script. The script takes fasta files aligned to the reference as input, and it calculates the noise for each position in the genome given a predefined set of mixing ratios between the input sequences. After manually adjusting the mixing ratios to 65%, 25% and 10% for the sequences “Major day 22”, “Minor day 0” and “Major day 0”, we found that the noise plot generated mimics the actual noise plot obtained after sequencing the sample.

### Cultivation of recombinant virus

Vero E6/TMPRSS2 cells (NIBSC #100978) were cultivated in complete Dulbecco’s Modified Eagle Medium (cDMEM) supplemented with 10% fetal bovine serum (FBS) and 1 mg/ml G418. In a biosafety level 3 (BSL3) laboratory, clinical samples collected from the patient were added to the cells at approximately 60% confluency in a T-25 flask for 1h at 37°C. The inoculum was subsequently removed and replaced with fresh viral culture medium (DMEM supplemented with 2% FBS, 100 units/ml penicillin, 100 μg/ml streptomycin and 25 mM HEPES). The infected cells were incubated for 3–4 days at 37 °C, and the supernatant was then diluted 1:1000 and passaged onto fresh cells for a second passage. After 3–4 more days the second passage of virus was harvested. Both the first and the second passage of the virus were sequenced.

### Fitness estimation

To estimate the fitness of the different virus strains, we identified the substitutions they carried at the amino acid level using Nextclade. Then, we connected those mutations with the fitness estimated from Bloom and Neher^26^. The fitness of each of the variants was computed as the sum of the fitnesses of the individual mutations present in the sample. If a sequence contained mutations absent in the fitness database, no fitness was assigned to that mutation.

### Study approval

The work has been carried out as part of the surveillance of infectious diseases at the Norwegian Institute of Public Health (NIPH). The patient was hospitalized and gave informed consent for samples being taken for diagnostics and for SARS-CoV-2 virus investigations and publication. The ethics committee/scientific department at the local hospital, Levanger Hospital, was consulted, and approval, was given to publish the results in current form. All methods were performed in accordance with relevant guidelines and regulations, the national infection control act and regulation on notification system for infectious diseases.

## Results

### Identification of a co-infection

**Fig. 1:**
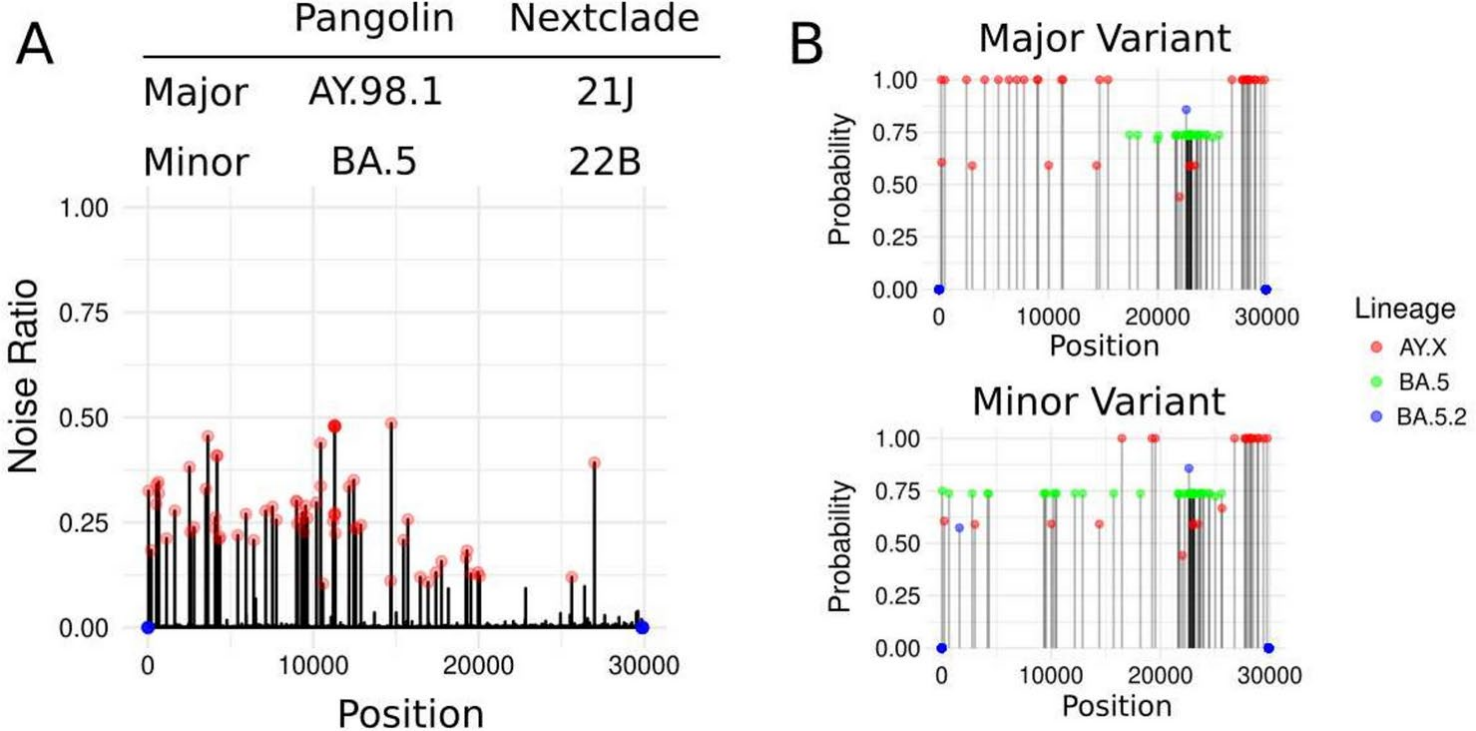
SARS-CoV-2 coinfection in a long-term infected COVID-19 patient. (**A**) Noise ratio of a sample and Pangolin and Nextclade classification of major and minor lineages extracted from the sample. The vertical lines represent the noise (i.e., variation in the sequence data) for each of the positions on the genome. The positions with a noise higher than 0.1were considered outliers and labeled with a red dot. The positions with a sequencing depth lower than 10 were labeled with a blue dot. (**B**) PrecFinder’s output showing Pangolin-lineage probability for the mutations present on the major and minor lineages for each mutation a probability of the harboring sequence belonging to a particular lineage is calculated (see “Methods” for details).

**Fig. 2:**
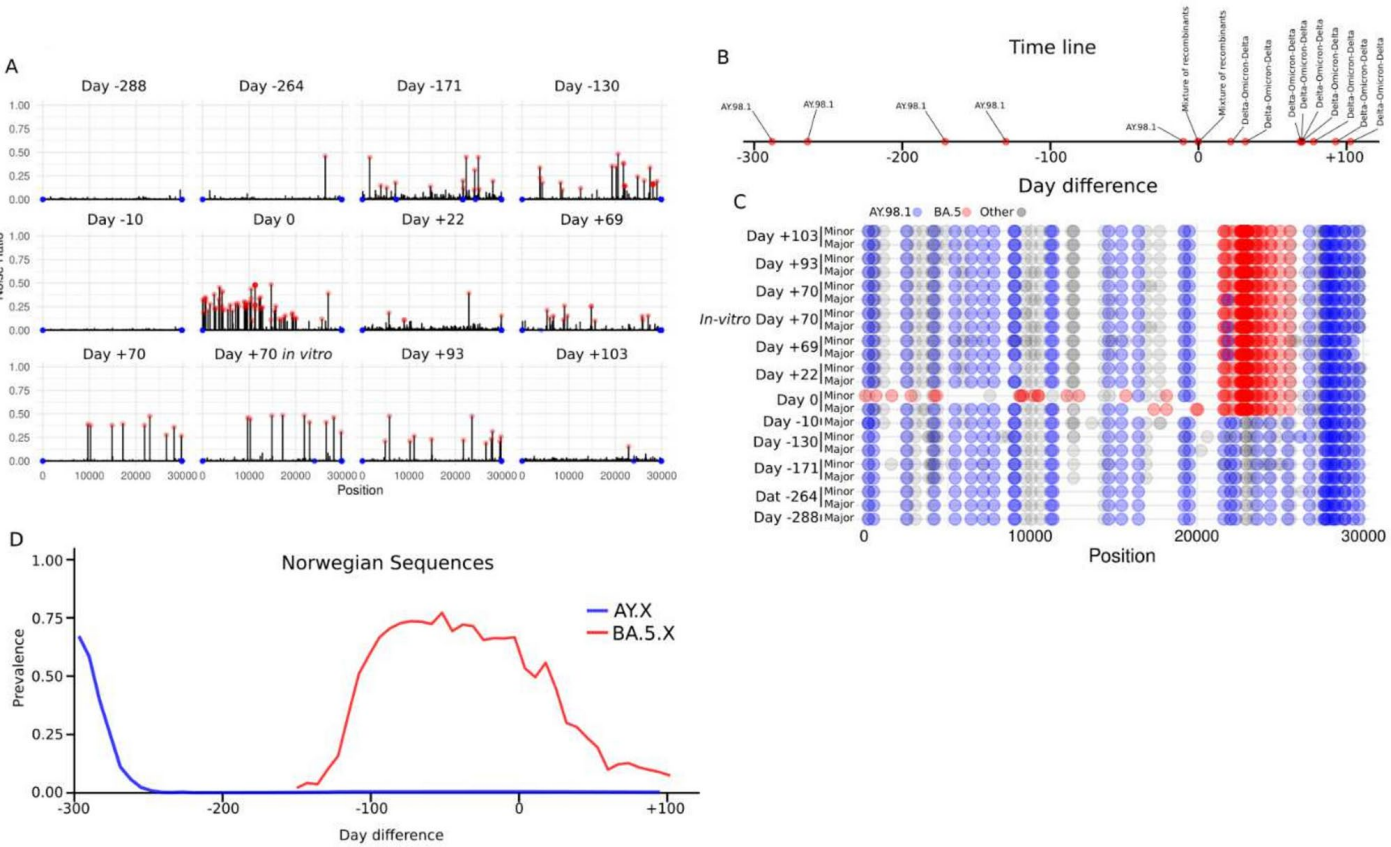
Identification of SARS-CoV-2 recombinants. (**A**) Noise ratio for all the consecutive samples analyzed. The noise outliers and missing positions were labeled with red and blue dots, as described in Fig. 1A. (**B**) Timeline of the sequences obtained from the patient together with the lineage that was obtained in each of them. (**C**) Nucleotide substitutions for all the sequences. The AY.98.1-specific mutations and BA.5-specific mutations were represented with blue and red dots respectively. Non-specific or unknown mutations were represented as gray dots. (**D**) Prevalence of BA.5 and AY.X lineages in Norway between days –300 and + 100.

As part of our SARS-CoV-2 surveillance role at the Norwegian Institute of Public Health (NIPH), we monitor the prevalence of the different viral variants in Norway. This surveillance is performed by sequencing representative SARS-CoV-2 samples gathered from different Norwegian locations. After sequencing the samples, the purity and consistency of the resulting sequence data are monitored. Through this quality control (see “Methods” for details), we identified a sample with high levels of noise, or sequence variation, after the mapping of the reads, which typically indicates either contamination or co-infection (Fig. 1A). After confirming that the sequence had good coverage and depth (Fig. S1A and B), we ruled out contamination and other sequencing artifacts as causes for the observation by repeating the entire analysis from RNA extraction, cDNA generation, PCR amplification, library preparation, to sequencing. The re-processed sample showed the exact same noise pattern (Figs. 1A and 2A “day 0”). This was the first and only time after sequencing and analyzing more than 60,000 samples where we clearly detected a pattern fully consistent with a mix of viruses in vivo*.*

Strikingly, the sequence variation was restricted mainly to the first two-thirds of the genome (Fig. 1A), which warranted further investigation. We attempted to re-create the genomic sequences of a potential major and minor variant in the sample (i.e., co-occurring strains varying in at least one nucleotide of high (> 50% of the reads) and low (< 50% and > 10% of the reads) abundances) (See “Methods” for details on the generations of the major and minor sequences) and we found that the major sequence was classified as a Delta variant (Pangolin lineage: AY.98.1/NextClade clade: 21 J) and the minor sequence as a variant of Omicron (BA.5/22B) (Fig. 1A and B).

### Identification of delta/omicron recombinant strains

Fine-grained mutation-profile analyses of the two strains (i.e., major and minor variants) showed that both sequences were recombinants. The major strain had AY.98-specific mutations in the first 15 Kb of the genome (flanked by G210T and G15451A) (Fig. 1B). Then, there was an 8 Kb region overlapping with the Spike gene that contains just BA.5 mutations (flanked by C17410T and C25584T). The minor strain, on the contrary, had only BA.5-specific mutations on the first 15.7 Kb of the genome flanked by the BA.5-specific C44T and C15714T mutations. Then, there was a 3 Kb region where there is a mixture of delta and omicron mutations. Next, there was a 4 Kb region covering the Spike gene where there are BA.5 mutations only (flanked by C21618T and C25584T BA.5 mutations) (Fig. 1B). The final part of the genome of both strains was identical and it carries only AY.98.1-specific mutations (Fig. 1B).

This suggests that the sample isolated on *day 0* was a mixture of at least two recombinant strains, one AY.98.1-BA.5-AY.98.1 recombinant and one BA.5-AY.98.1 recombinant.

Two independent recombinant-detection tools, sc2rf and PrecFinder, classified both strains as recombinants (Fig. 1B, Fig. S1C and D).

### Virus evolution in an immunocompromised long-term infected COVID-19 patient

Driven by these results, we became interested in tracing the sample’s origin, and found that it had been obtained from a long-term infected COVID-19 patient. Since the patient was already being monitored at the hospital, we could obtain five additional samples from the hospital collected from 288 days before the *day 0* sample and up to 10 days before (Fig. 2A and B). All these samples had much lower levels of noise compared to *day 0*, suggesting that the patient was only infected by a single viral strain at these time points. Some degree of noise was observed on *day -171* and *day -130*), but analysis of any major and minor strains in these samples, as well as all the other samples taken before *day 0*, showed only AY.98.1 strains without any BA.5-specific mutations (Fig. 2C). This indicates that the recombination events happened sometime in the ten days before *day 0.* The dominating lineages in Norway between day –130 and day 0 are consistent with the hypothesis that the patient was originally infected with an AY.98.1 virus and later co-infected with a BA.5 (Fig. 2D and Fig. S1E). To gain information on the relative fitness of the two recombinants, we decided to follow up the patient to see the viruses competing in vivo. We collected five extra samples from the patient at 22, 69, 70, 93 and 103 days after *day 0* (Fig. 2A and B). We found that the new samples had low noise levels, consistent with the presence of just a single lineage (Fig. 2A). Indeed, when we extracted major and minor variants from these new samples, we found that all of them belonged to a new recombinant lineage. This survivor lineage was the recombinant with an AY.98.1 backbone and a BA.5 Spike. We noticed that the lineage was similar but not identical to the major lineage found in the sample collected on *day 0* (Fig. 2C).

By looking at the mutation profiles (Fig. 2C) together with the noise across the genome (Fig. 2A) it seems unlikely that the minor lineage found on *day 0* was the one that outcompeted the other lineages. We therefore hypothesized two scenarios that could lead to the results observed 22 days after *day 0*. (i) The two recombinant lineages found on the *day 0* recombined again, so that the one that became dominant afterward lost four BA.5-specific mutations (i.e., C17410T, A18163G, C19955T, and A20055G) and acquired three AY.98.1-specific mutations (i.e., C16466T, C19220T, C19524T). (ii) Alternatively, in the sample taken on *day 0* there was indeed a mixture of at least three recombinants: a BA.5-AY.98.1 similar to the minor variant on *day 0*, a AY.98.1-BA.5-AY.98.1 similar to the major variant on *day 0*, and another AY.98.1-BA.5-AY.98.1 recombinant similar to the major variant present on the sample taken 22 days after *day 0*. A mixture of three such recombinants with approximate ratios 65%, 10% and 25% respectively, would produce a noise pattern like the one observed on day 0 (Fig. S1F versus Fig. 2A *day 0*). Although it is impossible to distinguish between these scenarios a posteriori, we believe that the second scenario is more plausible. Anyway, both scenarios require multiple recombination events suggesting that recombination between different SARS-CoV-2 lineages may occur frequently during co-infection.

### Cultivation of the recombinant lineage

To investigate the ability of the recombinant strain to propagate in vitro, we cultivated the virus extracted from the sample taken 70 days after *day 0*. We found that the virus was able to replicate and that after two passages, the sequence was the same as the original recombinant (Fig. 2C, *“day* + *70 in-vitro”*). Interestingly, we found that all the noisy positions present in the original sample were also noisy in the cultured samples (Fig. S1G), suggesting that the sample contained a mix of strains with different mutations at these positions. However, none of them seemed to provide a strong fitness advantage, at least in vitro.

### Fitness advantage of the recombinant

**Fig. 3:**
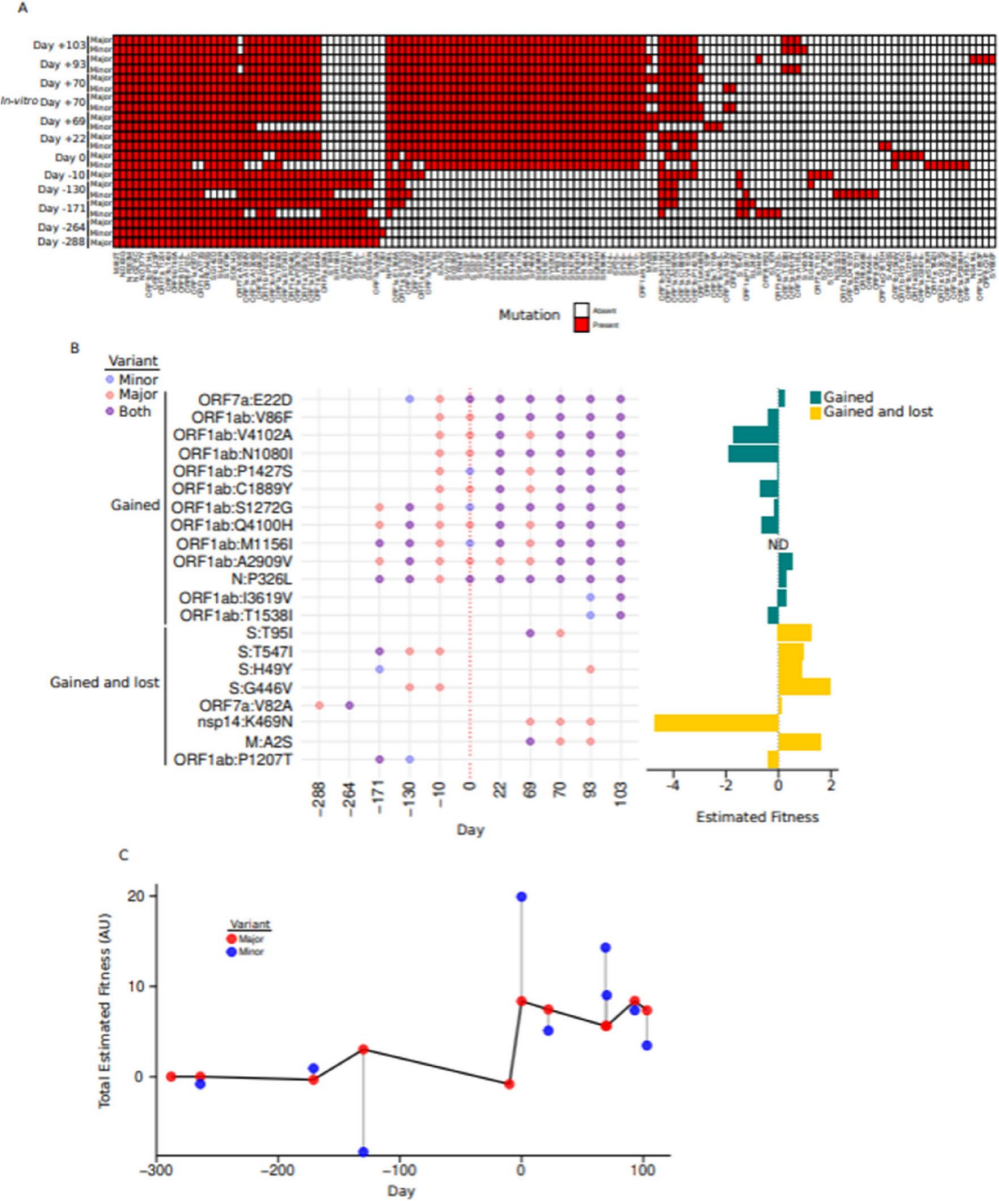
Fitness calculation. (**A**) Amino acid substitutions for all the sequences. (**B**) Mutations gained and or lost during the infection. The mutations present on the major and minor lineages were represented as red and blue dots respectively. Purple dots represent mutations present in both minor and minor lineages. The fitness associated with each of the mutations was obtained from the calculations performed by Bloom and Neher (2023) and represented as a barplot on the right. The fitness of mutations gained during the infection were represented with green bars and the fitness of the mutations *gained-and-lost* were represented with yellow bars. (**C**) Line and scatter plot of the overall fitness calculated for each sample. The individual fitnesses associated for each mutation present in a sample were added. The total fitness of the major lineages and minor lineages were represented with red and blue dots, and the line connects the fitness of the major lineages.

We hypothesized that the competition of two similar viruses inside a patient would be the perfect arena to infer which mutations would provide fitness advantages in vivo. After excluding the mutations gained or lost because of the recombination on *day 0*, we found 21 mutations with presence/absence patterns that suggested they had been gained and/or lost during the evolution of the virus within the patient (Fig. 3A and Fig. S1H). We found 13 mutations that were incorporated into the genome at some point during the infection (Fig. 3B), and we found eight mutations that were gained and then subsequently lost some time after.

Then, we analyzed the epidemiological fitness difference associated with these 21 mutations by connecting them with the fitness differences calculated by Bloom and Neher^26^. Surprisingly, we found that seven of the thirteen mutations gained and fixated into the genome are estimated to yield a negative fitness difference; conversely, six of eight mutations gained and lost had positive fitness differences.

Finally, we estimated the epidemiological fitness of all the variants identified by adding the individual epidemiological fitness associated with each of the mutations present in the viral genomes. We found that the major change in fitness in the dominant virus (i.e., the major variant) happened after the recombination event (Fig. 3C). Interestingly, we found that although the estimated fitness for some of the minor variants was higher than the fitness for the major one, including the minor variant on *day 0* (i.e., BA.5-Delta), those minor variants were outcompeted. This suggests that the epidemiological fitness estimated might not reflect the actual fitness of the virus to proliferate inside a patient.

## Discussion

Homologous recombination in coronaviruses is thought to occur when the enzyme RNA-dependent RNA polymerase (RdRp) separates from one RNA template while keeping the nascent RNA and then continues building the strand at the same position using a different template molecule^7^. Although coronaviruses have evolved to use recombination as part of their replication processes to produce a pool of recombined RNA molecules, the role of this viral molecular mechanism in generating novel recombinant lineages remains uncertain.

To our knowledge, this is the first report of a recombinant SARS-CoV-2 virus between the Omicron BA.5 and Delta AY.98 lineages and the first time that we have witnessed consecutive sequencing snapshots of the competition of several SARS-CoV-2 lineages in one infected individual. Although other studies have found recombinant viruses in sequential samples acquired from long-term infected patients^8^, this is the first time that we have obtained and analyzed samples in which at least two recombinant lineages were competing with each other in vivo. Moreover, we have developed and released a set of tools to detect and analyse this type of events in the future (i.e., Precfinder, NoisExtractor, Co-infection detection tool).

The results of this study reveal the emergence of a recombinant virus with an AY.98.1 backbone and a BA.5 Spike gene isolated from a long-term infected COVID-19 patient in Norway. The most likely scenario for this recombinant to arise is that, while at the hospital, the long-term patient infected with an AY.98.1 virus came in contact with another person infected by a BA.5 virus, leading to a co-infection and that shortly after the two lineages recombined. The recombined strain that eventually became the dominant strain in the patient probably arose within 10 days prior to the first detection of the recombinant lineage. However, our observations suggest that there were multiple recombination events within the patient, both between the Omicron and Delta variants, but also secondary events between different recombinants. These results suggest that recombination can occur frequently during co-infection, and they highlight the importance of close monitoring and early detection of such events.

Moreover, our findings suggest that several recombinant viruses may have been competing in the patient. And the fact that some apparently harmful mutations were retained over time, while beneficial mutations were lost, suggests that the evolution of the virus within the patient might be affected by clonal interference, a phenomenon where beneficial mutations may disappear from a population because of competition between sub-variants carrying different mutations^27^. By analyzing the epidemiological fitness associated with each of the observed mutations in the viral population in the long-term infected patient, we found that recombination had a significant impact on the fitness increase of the virus. Indeed, the fitness gained due to mutations acquired or lost during the infection seems to be lower than the fitness gained because of the incorporation of an Omicron Spike into a Delta backbone via recombination. Recombination in betacoronaviruses may therefore serve as a powerful mechanism to overcome clonal interference and ensure mixing of genetic material between lineages. Clonal interference is strongest in asexual organisms or when there is a strong linkage disequilibrium, but recombination might serve to overcome clonal interference. Indeed, one hypothesis that might explain the success of RNA viruses is that the high recombination rates in RNA-viruses might help them to overcome the burdens of clonality.

However, it is possible that our fitness estimations differ from the actual fitness of the virus for three reasons. First, the database that we used to estimate the fitness was constructed using epidemiological data gathered from viral databases, and it is possible that the mutations important for the fitness of the virus at the population level differ from the mutations important for its transmission between cells within the patient. This might be especially relevant for patients with a weakened immune system unable to clear the infection for months. Second, when we computed the overall fitness of each lineage, we did not account for epistatic relationships between mutations, and it is possible that genetic interactions between mutations become important determinants for the overall fitness of the virus. Third, the fitness associated with deletions of amino acids was not considered since the dataset does not have information about the fitness changes due to sequence deletions or insertions.

Therefore, further research is needed to investigate the potential implications of the mutations gained by the virus during its evolution within the patient (i.e., ORF7a:E22D, ORF1ab:V86F, ORF1ab:V4102A, ORF1ab:N1080I, ORF1ab:P1427S, ORF1ab:C1889Y, ORF1ab:S1272G, ORF1ab:Q4100H, ORF1ab:M1156I, ORF1ab:A2909V, N:P326L, ORF1ab:I3619V, ORF1ab:T1538I) in terms of fitness, transmissibility, virulence, and vaccine effectiveness.

## Conclusions

The identification of recombinant viruses in a long-term infected COVID-19 patient raises questions about the potential for similar events to occur in other patients and populations, as well as the implications for ongoing efforts to control the spread of the virus.

Our findings highlight the importance of continued surveillance and monitoring of SARS-CoV-2 genomes, particularly in high-risk populations such as long-term infected immunocompromised COVID-19 patients, to detect and respond to potential recombination events and other evolutionary changes in the virus. These patients are possibly one of the most probable sources for novel recombinant SARS-CoV-2 variants.

Overall, our study provides important insights into the genetic diversity and evolution of SARS-CoV-2 and underscores the need for ongoing research and surveillance efforts to better understand and combat this global health threat.

## Supplementary Information

Supplementary Figure S1.

Supplementary Legends.

## Data Availability

Sequences in fastq and fasta format are stored in the European Nucleotide Archive (ENA) under the Project ID PRJEB71327.

## Abbreviations

SARS-CoV-2: Severe Acute Respiratoy Syndrome coronavirus-2
ENA: European Nucleotide Archive
NIPH: Norwegian Insitute of Public Health
