# Supplementary material for: Recombinant SARS-CoV-2 Delta/Omicron BA.5 emerging in an immunocompromised long-term infected COVID-19 patient
Source: Sci Rep. 2024 Oct 28;14:25790. doi: 10.1038/s41598-024-75241-3 (PMC11519929; doi:10.1038/s41598-024-75241-3)
Supplement: Supplementary file 1 — Supplementary Figure S1. [file 41598_2024_75241_MOESM1_ESM.pdf]

# Figure S1

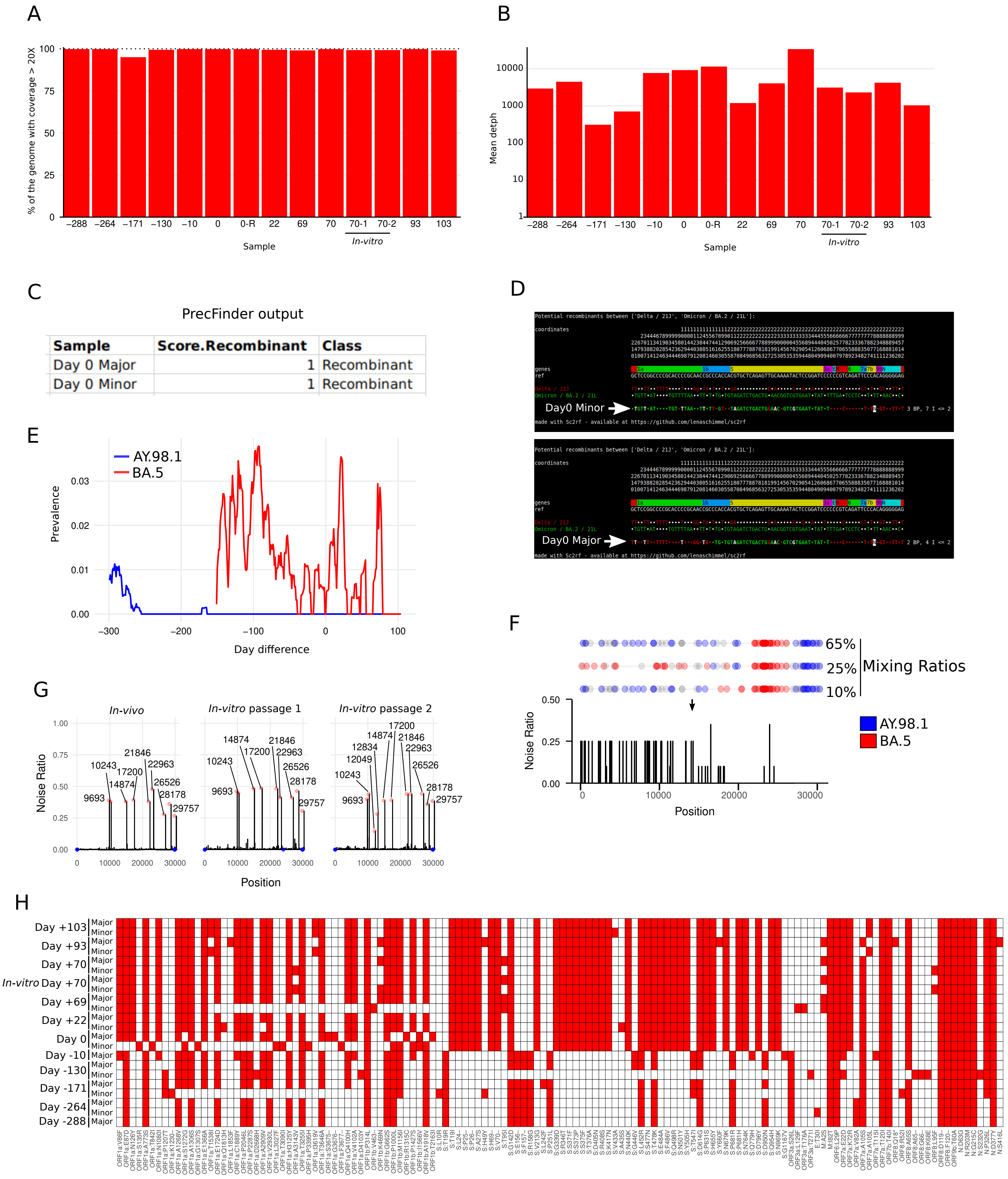

A
% of the genome with coverage > 20X
100
75
50
25
0
−288 −264 −171 −130 −10 0 0-R 22 69 70 70-1 70-2 93 103
In-vitro
Sample
B
Mean depth
10000
1000
100
10
1
−288 −264 −171 −130 −10 0 0-R 22 69 70 70-1 70-2 93 103
In-vitro
Sample
C
PrecFinder output
Sample | Score.Recombinant | Class
Day 0 Major | 1 | Recombinant
Day 0 Minor | 1 | Recombinant
D
Potential recombinants between ['Delta / 21J', 'Omicron / BA.2 / 21L']:
coordinates
genes
ref
Delta / 21J
Omicron / BA.2 / 21L
Day0 Minor
made with Sc2rf - available at https://github.com/lenaschimmel/sc2rf
Potential recombinants between ['Delta / 21J', 'Omicron / BA.2 / 21L']:
Day0 Major
made with Sc2rf - available at https://github.com/lenaschimmel/sc2rf
E
AY.98.1
BA.5
Prevalence
0.03
0.02
0.01
0.00
−300 −200 −100 0 100
Day difference
F
65%
25%
10%
Mixing Ratios
Noise Ratio
0.50
0.25
0.00
0 10000 20000 30000
Position
AY.98.1
BA.5
G
In-vivo
In-vitro passage 1
In-vitro passage 2
Noise Ratio
1.00
0.75
0.50
0.25
0.00
9693
10243
12049
12834
14874
17200
21846
22963
26526
28178
29757
0 10000 20000 30000
Position
H
Day +103
Day +93
Day +70
In-vitro Day +70
Day +69
Day +22
Day 0
Day -10
Day -130
Day -171
Day -264
Day -288
Major
Minor
