# Supplementary material for: Recombinant SARS-CoV-2 Delta/Omicron BA.5 emerging in an immunocompromised long-term infected COVID-19 patient
Source: Sci Rep. 2024 Oct 28;14:25790. doi: 10.1038/s41598-024-75241-3 (PMC11519929; doi:10.1038/s41598-024-75241-3)
Supplement: Supplementary file 2 — Supplementary Legends. [file 41598_2024_75241_MOESM2_ESM.docx]

**Figure S1.**

Recombinant viruses' confirmation. **A.** Whole genome coverage of all the samples included in this study. **B.** Average sequencing depth of all the samples included in this study. **C.** Classification of the major and minor sequences obtained on day *0* according to PrecFinder. **D.** Classification of the major and minor sequences obtained on day 0 according to sc2rf. **E.** Prevalence of AY.98.1 and BA.5 lineages during the time period of the study **F.** Simulated noise of a scenario in which three distinct recombinants (i.e. Major day 22, Minor day 0 and Major day 0 with ratios 65%, 25% and 10% were mixed “in silico”. **G.** Noise ratio of the sample obtained from the patient on day 70 (left) and the extracted virus cultivated for one (middle) or two passages (right). The noise outliers and missing positions were labeled with red and blue dots, as described in Fig. 1A. The nucleotide positions for the bases with high noise were also labeled. **F**. Amino acid substitutions for all the sequences ordered by its genomic location.
